# Beyond the Pass Mark: Accuracy of ChatGPT and Bing in the National Medical Licensure Examination in Japan

**DOI:** 10.31662/jmaj.2023-0043

**Published:** 2023-09-20

**Authors:** Yuki Kataoka, Sachiko Yamamoto-Kataoka, Ryuhei So, Toshi A. Furukawa

**Affiliations:** 1 Department of Internal Medicine, Kyoto Min-iren Asukai Hospital, Kyoto, Japan; 2 Scientific Research Works Peer Support Group (SRWS-PSG), Osaka, Japan; 3 Section of Clinical Epidemiology, Department of Community Medicine, Kyoto University Graduate School of Medicine, Kyoto, Japan; 4 Department of Healthcare Epidemiology, Kyoto University Graduate School of Medicine/School of Public Health, Kyoto, Japan; 5 Department of Health Informatics, Kyoto University Graduate School of Medicine/School of Public Health, Kyoto, Japan; 6 Department of Psychiatry, Okayama Psychiatric Medical Center, Okayama, Japan; 7 Department of Health Promotion and Human Behavior, Kyoto University Graduate School of Medicine/School of Public Health, Kyoto, Japan

**Keywords:** Large Language Model, ChatGPT, Evidence-based Medicine

## Introduction

The evolution of information technology has played a crucial role in the history of evidence-based medicine (EBM). In the 1990s, the widespread use of the Internet and personal computers changed clinical problem-solving from seeking expert opinions to conducting searches and reading/critically appraising articles ^(1)^. In the 2000s, search engine development enabled more people to easily search for medical information ^(2)^. Since its release in November 2022, ChatGPT, an easy-to-use chat-style large language model (LLM), has helped searching medical knowledge through casual conversations ^(3), (4)^. Since February 2023, the integration of ChatGPT into the search function of Bing has been available to anyone who wanted to use it ^(5)^.

A chat system using LLM can answer questions. Thus, by providing evidence in immediate response to clinical questions, the chat system could speed up the process of health professionals searching for and critically appraising evidence. However, the accuracy of chat-based information-retrieval technologies has not been thoroughly evaluated yet. In this study, we investigated the accuracy of ChatGPT and Bing using its responses from the National Medical Licensure Examination in Japan. Particularly, the reasons for the incorrect responses of LLM were probed by medical professionals.

## Methods

We conducted a cross-sectional study. We used questions from the 116^th^ National Medical Licensure Examination in Japan conducted in February 2022 ^(6)^. ChatGPT is a large language model trained on data up to 2021, whereas Bing employed a subsequent version of ChatGPT named GPT-4. The details of both models remain undisclosed.

All questions were multiple-choice and written in Japanese. From a total of 400 questions, we excluded 100 questions with pictures and five inappropriate questions that could not be answered ^(7)^. We did not exclude two inappropriate questions for being too difficult for medical students. We also fixed some garbled Kanji characters. The tabular choices were rewritten in text format. We have reposited the raw questions and answers to the spreadsheet in the Open Science Framework (https://osf.io/jq7c8/).

To test ChatGPT, we used the ChatGPT PLUS default mode from February 3 to 15, 2023. One researcher manually entered, “I am going to give you quizzes from the National Medical Licensure Examination in Japan. Select one correct choice unless otherwise stated.” Each question was entered into a questionnaire. If ChatGPT selected more options than the question was set up for, we additionally asked it to “choose only one appropriate question” once.

To test Bing, we used Bing chat from February 16 to 28, 2023. One researcher manually entered, “I will ask you quizzes based on National Medical Licensure Examination in Japan. Please exclude the following websites from your answers: https://www.medu4.net/., .https://medical-illustration.club/, https://www.mhlw.go.jp/seisakunitsuite/bunya/kenkou_iryou/iryou/topics/, and https://medical.nikkeibp.co.jp/. You do not need to provide explanations for each answer choice but please list the websites that you used as references. Do you understand?” We subsequently encoded all questions. The aforementioned websites to be excluded presented correct answers. Bing did not provide revised answers, although we commented that the number of answers was incorrect. Hence, we did not ask any follow-up questions.

To evaluate the incorrect answers provided by Bing, a board-certified specialist in internal medicine (KY) categorized them into the following four categories: misinterpretation of the meaning of the question statement, incorrect diagnosis, correct diagnosis with wrong information, and incorrect information. Another author (SYK) confirmed the categorization by KY. Disagreements were resolved through discussion.

## Results

From the 295 candidate questions, we excluded 14 in which Bing referred to the website where the answer was posted. Finally, we evaluated 281 questions, and answers using ChatGPT, and Bing. The ChatGPT and Bing scores for the compulsory and other questions are presented separately in Table 1. Bing was correct in 219 (78%) questions. All incorrect answers in ChatGPT were attributed to “wrong information.” ChatGPT did not provide a reasoning process or a clinical diagnosis. Table 2 shows the reasons behind the incorrect answers provided by Bing.

**Table 1.** Scores by Large Language Models.

Characteristic	Compulsory questions, N = 81^1^	Others, N = 200^1^	Total, N = 281
Bing score	70 (86%)	149 (74%)	219 (78%)
ChatGPT score	34 (42%)	72 (36%)	106 (38%)

^1^N (%)

**Table 2.** Reasons for Wrong Answers by Bing.

Characteristic	Normal questions, N = 31^1^	Clinical questions, N = 31^1^
Misinterpretation of the meaning of the question statement	10 (32%)	7 (22.6%)
Wrong diagnosis	0 (0%)	3 (9.7%)
Wrong information with a correct diagnosis	0 (0%)	4 (13%)
Wrong information	21 (68%)	17 (55%)

^1^N (%)

## Discussions

Bing outperformed ChatGPT in terms of scores. To pass Japan’s National Medical Licensure Examination, medical students need a score of at least 80% for compulsory questions and 70% for other questions. The discrepancy in performance between the two LLMs possibly results from the differences in the amount of training data used for learning. Bing answered the questions for which it had answers. As for important considerations, Bing may have been trained on answers that were not cited. Thus, Bing might have had prior knowledge of the questions and correct answers, possibly leading to overestimating medical inference performance. Most incorrect answers were due to wrong information that could not be determined as incorrect without prior knowledge.

The accuracy of ChatGPT herein was lower than that in prior studies using the United States Medical Licensing Examination ^(3), (4)^. The limited amount of Japanese language data may have affected the ability of ChatGPT to correctly answer medical questions in Japanese translation ^(8)^. Considering language is crucial when applying the LLM to other language translations.

Bing has an error proportion of approximately 20%, and users cannot determine the correctness of an answer based solely on its wording. Therefore, health professionals should critically appraise the references cited by Bing; the process is not significantly different from searching for literature using existing search engines. Health professionals should avoid applying the LLM answers in clinical practice without critical appraisal. Further research is warranted to determine whether using LLM can reduce the time required to find relevant literature compared to existing search methods.

In conclusion, Bing is sufficiently accurate to pass Japan’s national medical licensing exam. However, the LLM answers cannot be used for clinical decision-making in its current form. Further research to investigate the potential of LLMs as search tools is warranted.

## Article Information

### Conflicts of Interest

Yuki Kataoka: None declared; Sachiko Yamamoto-Kataoka: None declared; Ryuhei So: RS has received research grants from the Japan Society for the Promotion of Science (JSPS); Ministry of Health, Labor, and Welfare, Japan; Japan Agency for Medical Research and Development; Osake-no-Kagaku Foundation; The Mental Health Okamoto Memorial Foundation; and Kobayashi Magobe Memorial Medical Foundation; and speaker’s honoraria from Otsuka Pharmaceutical Co., Ltd., Nippon Shinyaku Co., Ltd. and Takeda Pharmaceutical Co., Ltd. outside the submitted work. RS also reports an employment position at CureApp Inc., which develops software as medical devices; Toshi A. Furukawa: TAF reports personal fees from Boehringer-Ingelheim, DT Axis, Kyoto University Original, Shionogi and SONY, and a grant from Shionogi, outside the submitted work; In addition, TAF has patents 2020-548587 and 2022-082495 pending, and intellectual properties for Kokoro-app licensed to Mitsubishi-Tanabe.

### Acknowledgement

The authors underwent paragraph-by-paragraph editing using ChatGPT. All authors reviewed and edited the final manuscript. The responsibility for the content of this article rests solely with the authors. The English editing costs were partly supported by a JSPS Grant-in-Aid for Scientific Research (grant number: 22K15664) to YK.

### Author Contributions

YK had full access to all data in the study and took responsibility for the integrity of the data and the accuracy of the data analysis. Data extraction: YK and SYK. Manuscript drafting: YK. Critical manuscript revision for important intellectual content: SYK, RS, and TAF. All authors gave their final approval for the version to be published and agreed to be accountable for all aspects of this study.

### Approval by Institutional Review Board (IRB)

Patient consent for publication: Not applicable.

Ethics approval: Not applicable.

Funding: Not applicable.
